# Draft Genome Sequences of Three Strains of *Acidithiobacillus* spp. Isolated from Acidic Cold Springs in a Freshwater Wetland

**DOI:** 10.1128/MRA.00090-21

**Published:** 2021-04-01

**Authors:** H. V. Parker, C. L. Marnocha

**Affiliations:** a Department of Biology, Niagara University, Lewiston, New York, USA; University of Delaware

## Abstract

We isolated three strains of *Acidithiobacillus* spp. from an acidic spring in a freshwater wetland. Here, we report the draft genomes of these three strains, which were obtained using Illumina-based sequencing technology.

## Abstract (precis)

We isolated three *Acidithiobacillus* sp. strains from an acidic spring in a freshwater wetland. Here, we report the draft genomes of these three strains, which were obtained using Illumina-based sequencing technology.

## ANNOUNCEMENT

The Iroquois National Wildlife Refuge in Basom, New York (43.125383N, 78.370095W), is home to emergent marsh and hardwood swamp and is notable for the presence of acidic cold springs (pH < 2) (1). We isolated three *Acidithiobacillus* strains from one of these springs. While acidithiobacilli are found in naturally acidic environments (2–4), most isolates come from acid mine drainage (AMD). To investigate how these populations might differ from those in AMD, we sequenced the genomes of our three *Acidithiobacillus* isolates.

Samples were collected approximately 15 cm beneath the surface of the spring pool using a sterile pipette. Samples were used to inoculate liquid ATCC 1353 medium to enrich for acidophilic sulfur-oxidizing bacteria. Enrichment cultures were streaked onto ATCC 1353 agar plates (3% agar), and single colonies were transferred to fresh liquid medium. All cultures were grown at room temperature at a pH of ∼4. We extracted DNA from liquid culture pellets using a DNeasy PowerSoil kit (Qiagen). The 16S rRNA genes were PCR amplified using GoTaq Green master mix (Promega, Madison, WI) according to the manufacturer’s instructions, with universal 27F and 1492R primers. Amplicons were purified using a QIAquick PCR purification kit (Qiagen) and sent for Sanger sequencing at Eurofins Genomics. We subjected the sequences to a search against the nonredundant database using BLASTn with default settings to determine taxonomy. We identify our strains as *Acidithiobacillus* sp. strains HP-2, HP-6, and HP-11.

Extracted DNA was sent to the Integrated Microbiome Resource (IMR) for whole-genome sequencing. The genomic library was constructed using the Illumina Nextera XT kit with 1 ng of DNA, dual indexed, and then run on a MiSeq system using 600-cycle v. 3 chemistry (300 + 300 bp) according to the manufacturer's instructions except that library cleanup and normalization were completed using the Just-a-Plate 96 PCR purification and normalization kit (Charm Biotech). Reads were paired, quality trimmed, and filtered within Geneious Prime using the BBDuk trimmer v. 1.0 (5). We used the Geneious *de novo* assembler v. 2019.0.3 on the “medium-low” setting, and contigs of >1,000 bp were used in subsequent analyses. Assemblies were assessed with CheckM v. 1.0.18 (6), and all isolates were determined to be 99.34% complete with 0.62% contamination. Average nucleotide identity (ANI) values were determined using FastANI v. 0.1.2 (7). Assemblies were submitted to NCBI for gene calling and annotation using the Prokaryotic Genome Annotation Pipeline (PGAP) v. 4.12 (8). Default parameters were used for all software unless otherwise specified. Detailed genome assembly statistics are provided in Table 1.

**TABLE 1:** Genome attributes of *Acidithiobacillus* HP isolates

Parameter	Data for strain:		
	HP-2	HP-6	HP-11
No. of reads	6,769,528	2,265,194	7,737,332
No. of contigs	48	52	113
*N*_50_ (bp)	169,287	144,660	48,172
Genome size (bp)	3,200,419	3,209,818	2,954,837
GC content (%)	52.95	52.96	52.88
No. of coding sequences	3,247	3,248	3,003
No. of 5S rRNAs	2	2	2
No. of 16S rRNAs	1	1	2
No. of 23S rRNAs	2	2	1
No. of tRNAs	46	46	47
Coverage (×)	321	209	199
ANI with HP-2 (%)	99.9449	94.9005	
ANI with HP-6 (%)	99.9501	94.7756	
ANI with HP-11 (%)	95.0033	95.0258	
GenBank accession no.	JACKZA000000000	JACKYZ000000000	JACKYY000000000
SRA accession no.	SRR10882953	SRR10882952	SRR10882951

We found a similar complement of genes in our isolates, compared with other Acidithiobacillus thiooxidans strains. A notable exception includes the presence of the *dnd* operon (*dndBCDE*) in the HP-2 and HP-6 genomes. The *dnd* operon is proposed to play a role in adaptation to extreme environments, enabling expanded growth ranges under extreme conditions (9) through phosphorothionate modification of the DNA (9, 10). The *dnd* operon is widespread in a diverse group of prokaryotes (11, 12) but is not known to be in any other acidithiobacilli. Overall, these draft genomes offer insight into the functional capacity of *Acidithiobacillus* strains from a naturally acidic environment.

### Data availability.

Assemblies and raw reads have been deposited in GenBank under the accession numbers given in Table 1 (BioProject accession number PRJNA599179).
